# Short‐Term Nociceptive Memory: Reduced Discriminability and Multiple Encoding Biases

**DOI:** 10.1002/ejp.70323

**Published:** 2026-06-30

**Authors:** Maud Frot, Caroline Perchet, Juliette Gelebart, Luis Garcia‐Larrea

**Affiliations:** ^1^ NeuroPain Lab/CRNL, Inserm U1028, CNRS, UMR5292, Université Claude Bernard Lyon 1 Lyon France

**Keywords:** delayed discrimination, nociception, pain memory, perceptual bias, short‐term memory

## Abstract

**Background:**

Distortions in pain memory carry important clinical implications, yet the processes underlying retention of nociceptive information remain incompletely understood. This study examined how the intensity of painful somatosensory inputs is maintained in memory over different intervals.

**Methods:**

Twenty‐five participants received pairs of nociceptive or non‐nociceptive electrical stimuli at varying inter‐stimulus intervals (3, 8, 13, 18 s), and judged whether the second stimulus was of higher, equal, or lower intensity than the first.

**Results:**

In accordance with Weber‐Fletcher Law, perceptual discriminability was lower in the nociceptive condition, leading to a decreased accuracy despite comparable confidence ratings. Memory performance declined with temporal delay similarly for both modalities. Accuracy increased when the second stimulus was more intense than the first. For nociceptive stimuli only, accuracy deteriorated disproportionately when the second stimulus was weaker, suggesting a directional encoding bias specific to pain. When errors occurred, participants overestimated the intensity of the second stimulus, a bias more pronounced for nociceptive stimuli. Such overestimation was linked to memory encoding and disappeared when participants rated stimuli without memory demand.

**Conclusions:**

Short‐term memory for nociceptive stimuli proved less accurate, more directionally biased, and more prone to overestimation than for non‐nociceptive inputs. While this performance gap is largely explained by reduced perceptual discriminability at pain intensities, modality‐specific distortions point to additional constraints imposed by nociceptive processing on memory encoding and maintenance.

**Significance:**

This study provides psychophysical evidence for differences in short‐term memory retention of nociceptive versus non‐nociceptive sensory input, with implications for understanding pain memory distortions in clinical contexts.

## 1 Introduction

While it is widely accepted that a correct understanding of pain memorisation mechanisms is crucial for a better comprehension of pain pathologies (Yi and Zhang 2011; McCarberg and Peppin 2019), studies addressing memory processes for nociceptive input are extremely rare. The clinical evaluation of patients' pain relief is commonly based on a comparison of current versus past pain, but such reports are most often inaccurate when compared to daily pain rates (Matera et al. 2003; Broderick et al. 2008; Giske et al. 2010; Daoust et al. 2017; Wikström et al. 2017). Pain memories are highly malleable and can be influenced by internal (Bąbel 2017) or external states, including misinformation (Urban et al. 2019). The emotional context surrounding the initial pain experience profoundly influences subsequent memory formation: high emotional arousal facilitates consolidation and amplifies the remembered pain intensity (Gedney and Logan 2004; Rocha et al. 2009; Terry et al. 2007; Kyle et al. 2016; Fischer et al. 2019), while positive affective states associated with initial pain can lead to its underestimation when remembered (Bąbel et al. 2015, 2018; Farley et al. 2019; Anunciação et al. 2022). These memory distortions occur consistently across healthy populations and clinical samples, influenced by multiple contextual factors (for reviews, see Erskine et al. 1990, Adamczyk et al. 2019).

Pain memory research faces methodological challenges, particularly in the parametric manipulation of long‐term memory processes. Working memory for pain offers a more tractable experimental framework, enabling precise quantification of performance maintenance, temporal decay patterns, and systematic comparison across stimulus modalities. The investigation of working memory holds particular theoretical significance given the partially overlapping neural networks underlying short‐term and long‐term memory systems (Fuster 1998, 2022; Vo et al. 2021); elucidating the mechanisms governing working memory processes may therefore provide critical insights into the neural substrates of pain memory more broadly. One of the most extensively validated approaches for examining short‐term memory is the matching‐to‐sample, also termed delayed discrimination task, which requires participants to maintain stimulus features in working memory before comparing them with subsequently presented test stimuli. Using thermal stimuli at noxious and non‐noxious intensities, Rainville et al. (2004) showed that working memory for thermal nociception undergoes rapid temporal degradation, with retention capacity declining significantly within seconds of stimulus offset. This rapid decay pattern raises important questions about the fundamental characteristics of nociceptive memory storage and retrieval processes that warrant systematic investigation.

The aim of the present study was to compare short‐term memory for nociceptive and non‐nociceptive brief somatosensory stimuli using a delayed discrimination task, focusing on both retention capacity—by varying interstimulus intervals (ISIs) between 3 and 18 s—and response reliability, assessed through subjective certainty ratings. Four research questions guided this investigation: (i) whether short‐term memory capacity differs between nociceptive and non‐nociceptive stimuli; (ii) how interstimulus interval duration affects retention performance; (iii) how stimulus modality influences subjective response certainty; and (iv) how relative stimulus intensity within stimulus pairs affects retention capacity and memory biases.

## 2 Methods

### 2.1 Subjects

Twenty‐five healthy subjects (23.8 ± 3.2 years, 12 women) participated in the study. Inclusion criteria included absence of neurological, psychiatric or chronic painful disorders, no treatment with painkillers, antidepressants or psychotropic drugs, and absence of memory impairments, as demonstrated by normal results to a memory span test (MEM III, Wechsler 1945, 2001) performed prior to the experiment. Each subject was informed of the procedure established in the experience, which was approved by the local Ethics Committee (CCPPRB 2015‐010B Léon Bérard‐Lyon).

### 2.2 Stimulations

Nociceptive and non‐nociceptive electrical stimuli were delivered on the right thumb of each subject through a Micromed (ENERGY) stimulator. Nociceptive and non‐nociceptive stimuli were delivered in different sessions, respectively through a concentric planar electrode (Walter Graphtek GmbH, Lübeck, Germany) and through ring electrodes. Stimuli consisted in both cases of monophasic constant current pulses of 100 μs duration delivered at nociceptive or non‐nociceptive intensities.

For each type of stimuli (nociceptive or not), “high” and “low” intensity levels were determined prior to the experiment, and tailored to each subject. It was ensured previous to the main experiment that these high and low intensities were such that (a) each participant could distinguish easily between them, and (b) they rated clearly above (at least 1.5 points on the VAS above the threshold) the pain threshold for the nociceptive modality, and clearly below for the non‐nociceptive one (at least 1.5 points on the VAS below the threshold). Once determined in a given subject, the two intensities in each modality were kept stable during the whole experiment.

The choice of brief electrical pulses (100 μs duration) was driven by several methodological considerations. First, electrical stimulation allows precise temporal control over stimulus onset and offset, which is critical for accurately manipulating inter‐stimulus intervals. Second, unlike prolonged thermal stimuli, brief electrical pulses minimize peripheral adaptation, habituation, or sensitization effects across the 256 total stimuli delivered throughout the experiment, ensuring stable perception. Third, when delivered through concentric planar electrodes, brief electrical pulses easily activate nociceptive Aδ fibres, providing a clearly pricking nociceptive stimulus.

### 2.3 Experimental Paradigm

Participants were comfortably seated in front of a computer screen. All ratings were done on visual analog scales (VAS) to discourage any numerical encoding of the sensation. We used visual analog scales with verbal anchors, which are widely accepted in pain research. Scales ranged from “slightly painful” to “extremely painful” for nociceptive shocks, and from “no sensation” to “strongly perceived” for non‐painful stimuli. Subjects rated their sensations on these VAS by clicking with the mouse button on a horizontal line displayed on the computer screen, with the left and right extremities showing the extreme minimal and maximal perceptions described above according to the type of stimulation (painful or non‐painful).

#### 2.3.1 Training Session

Perceptual stability was assessed by having participants rate 14 consecutive stimuli (7 of each intensity, randomly delivered) prior to the main experiment. Participants were included only if they consistently rated high‐intensity stimuli as more intense than low‐intensity stimuli in ≥ 85% of trials, and showed significant group‐level discrimination between intensities (independent samples *t*‐tests).

#### 2.3.2 Main Experiment

**FIGURE 1:**
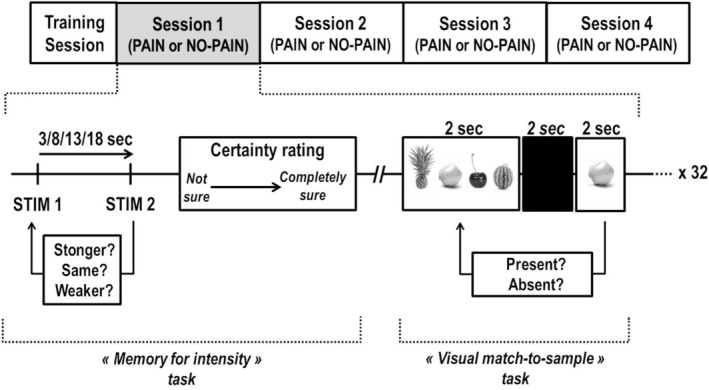
Experimental paradigm. During a training session, subjects had to rate on a VAS their sensation for 14 stimuli (7 of each intensity). During the main experiment, each trial comprised a somatosensory and a visual memory task. The somatosensory task was a ‘memory for intensity task’, where two electrical pulses were delivered, separated by a variable interval of 3–18 s. Subjects had to rate the intensity of the second stimulus relative to the first (equal, weaker, stronger), and indicate the subjective certainty of their responses. Then, a visual matching‐to‐sample test was performed, where subjects indicated if a visual image pertained or not to a set of four items presented before. A session was composed of 32 trials (somatosensory + visual), and each subject performed two painful blocks and two non‐painful blocks.

After ensuring that participants were able to provide stable and reproducible ratings, the main experiment was composed of 4 sessions, two nociceptive and two non‐nociceptive, presented in random order. Each session consisted of 32 trials, and each trial was composed of a ‘memory for intensity’ task, followed by a ‘visual match‐to‐sample task’ (Figure 1). The inter‐trial interval (time between the end of one trial and the start of the next) was approximately 20 s. Sessions were separated by breaks of at least 5 min to minimize cumulative peripheral effects such as sensitization or habituation.

In the ‘memory for intensity’ *task*, subjects received pairs of stimuli of the same modality (either nociceptive or non‐nociceptive), separated by a random interval of 3, 8, 13 or 18 s. Each pair consisted of one of four possible intensity combinations: low–low, low–high, high–low, or high–high. Participants were instructed as follows: “You will receive two electrical stimulations separated by a short delay. Your task is to judge whether the second stimulus was of higher, equal, or lower intensity compared to the first stimulus”. Since participants had no direct access to the physical stimulus intensity (in mA), their judgements necessarily relied on their memory of the subjective experience elicited by each stimulus. Performance was assessed based on response accuracy, defined as the proportion of correct answers (correct response rate). Participants also rated their subjective certainty using a visual analog scale (VAS), indicating their confidence by clicking on a horizontal line displayed on the screen, anchored from “not sure” to “completely sure.” These responses were then transformed into numerical values, with the endpoint labelled “completely sure” set to 100%, resulting in a quantified certainty rate.

In the ‘visual match‐to‐sample’ task, 4 neutral pictures depicting fruits, vegetables or everyday objects were presented on the screen for 2 s, followed by 2 s' black screen. Then, a single item appeared and the subjects had to decide whether it pertained or not to those previously presented. This visual task was presented immediately after each somatosensory trial, to erase possible residual memory traces from the ‘memory for intensity’ task, and to ensure that comparison between each pair of somatosensory stimuli was performed exclusively inside a single pair, and not influenced by past comparisons (Figure 1).

### 2.4 Analyses

All statistical analyses were performed with JASP software and significance level was set at *p* < 0.05. For all analyses of variance, assumptions of normality and sphericity were examined; for factors with more than two levels, sphericity was tested using Mauchly's test, and Greenhouse–Geisser corrections were applied when necessary. Post hoc pairwise comparisons were performed with Bonferroni correction in the case of significant main effects.

#### 2.4.1 Intensity Rating Test (Training Session)

Rating averages were calculated for each subject and for each intensity delivered (2 painful, 2 non‐painful). A Student's test was then applied to compare the overall ratings between low and high intensities for painful and non‐painful stimuli, in the whole group.

#### 2.4.2 “Memory for Intensity” Task

Normality was assessed using the Shapiro‐Wilk test. The results indicated that the distributions of both accuracy and certainty scores did not significantly deviate from normality in either condition (Accuracy_Pain: *W* = 0.96, *p* = 0.36; Accuracy_NoPain: *W* = 0.97, *p* = 0.66; Certainty_Pain: *W* = 0.97, *p* = 0.68; Certainty_NoPain: *W* = 0.96, *p* = 0.48), thus justifying the use of parametric analyses.

##### 2.4.2.1 Effect of Stimulus Mode

To assess differences in participants' performance and confidence across conditions, paired‐sample *t*‐tests were conducted on both accuracy (percentage of correct responses) and certainty ratings, comparing the pain and no‐pain conditions. In addition, Pearson correlation analyses were performed separately for each condition to investigate the relationship between accuracy and certainty, in order to determine whether participants' subjective confidence was aligned with their actual performance in the pain and no‐pain contexts.

##### 2.4.2.2 Effect of Inter‐Stimulus Interval (ISI)

To investigate the effects of inter‐stimulus intervals and condition on task performance, we conducted two separate repeated‐measures analyses of variance (ANOVAs) on correct response rates and certainty ratings, each including two within‐subject factors: inter‐stimulus interval (3 s vs 8 s vs 13 s vs 18 s) and condition (Pain vs No Pain).

##### 2.4.2.3 Effect of the Direction of Intensity Change

To examine the influence of stimulus pair composition on task performance, we conducted two separate repeated‐measures ANOVAs, one for correct response rates and one for certainty ratings. Each ANOVA included two within‐subject factors: **Condition** (Pain vs. No Pain) and **Pair Type**, corresponding to the combination of stimulus intensities within each pair (Low–Low, Low–High, High–Low, High–High).

##### 2.4.2.4 Analysis of Errors: Over‐ Versus Under‐Estimates

Error types were analysed by comparing the overestimation and underestimation rates with a repeated measures ANOVA with error type (underestimate vs. overestimate) and stimulation type (Pain vs. No‐Pain) as factors. Over‐ and under‐estimation rates were calculated relative to the total number of responses. The changes in overestimation rates were also analysed as a function of ISI (3 vs. 8 vs. 13 vs. 18 s) and according to the intensity of the first stimulus in a pair (First High vs. First Low).

##### 2.4.2.5 Control for Perceptual Difference Magnitude

To assess whether differences in discriminability between high and low intensities could explain memory performance, we first calculated, for each participant, the perceptual difference magnitude (ΔVAS) by subtracting the mean VAS rating for low‐intensity stimuli from that of high‐intensity stimuli, separately for each modality. We then conducted a linear mixed‐effects model (LMM) predicting correct response rates. Condition (Pain vs. No‐Pain), ΔVAS, and their interaction were included as fixed effects, with random intercepts for participants.

#### 2.4.3 Visual “Match‐To‐Sample” Memory Task

Correct recognition rates were computed for each session, together with a global mean across all sessions. Performances were compared across sessions using one‐way repeated measures ANOVA with session (session 1 vs session 2 vs session 3 vs session 4) as factor.

## 3 Results

### 3.1 Intensity Rating Test (Training Session)

**FIGURE 2:**
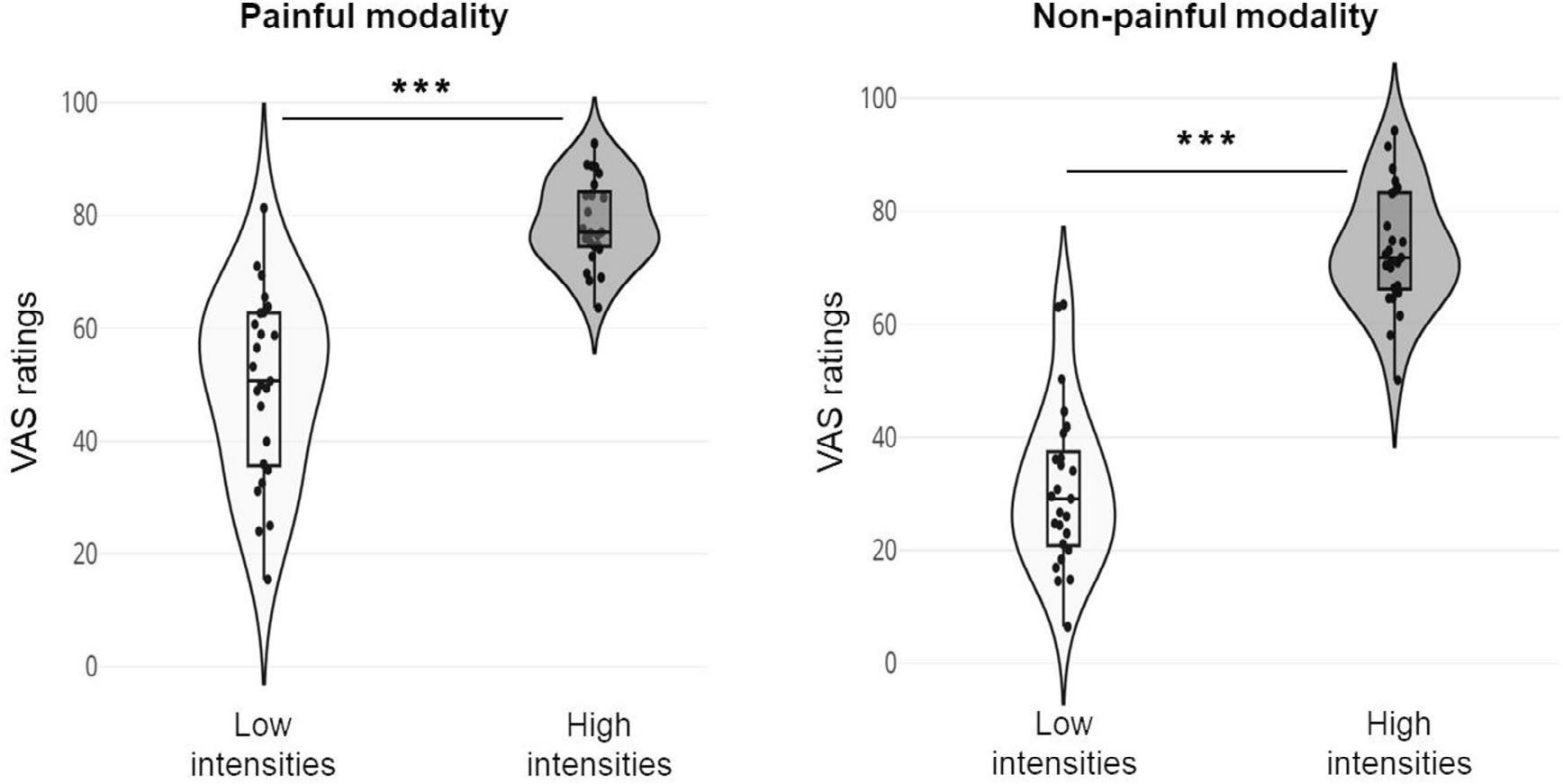
Violin plots displaying the distribution of VAS (Visual Analog Scale) ratings for low and high intensity stimuli in both painful and non‐painful modalities. ***p < 0.001.

In the preliminary training test, subjects correctly distinguished the two intensities chosen for each modality (painful and non‐painful, Figure 2), both at the group level (difference of ratings: *t*(24) = −9.6, *p* < 0.001, Cohen's *d* = −1.9 for nociceptive stimuli; *t*(24) = −15.1, *p* < 0.001, Cohen's *d* = −3.02 for non‐nociceptive; mean VAS differences: 29.7/100 for nociceptive stimuli, 42.4/100 for non‐nociceptive modality) and at individual level (100% of subjects distinguished precisely the two intensities). No statistical comparison was made between ratings of painful and non‐painful intensities because the visual analog scales were different for the two stimulus modalities (see Methods).

### 3.2 “Memory for Intensity” Task

A one‐sample *t*‐test revealed that participants' performances in the task were significantly above chance level (Pain: *t*(24) = 8.22, *p* < 0.001, Cohen's *d* = 1.74; No pain: *t*(24) = 15.22, *p* < 0.001, Cohen's *d* = 3.04). Since participants had three response options (higher, equal, or lower intensity), chance performance corresponds to 33.3% (1/3) correct responses if participants were responding randomly.

#### 3.2.1 Effect of Stimulus Mode

**FIGURE 3:**
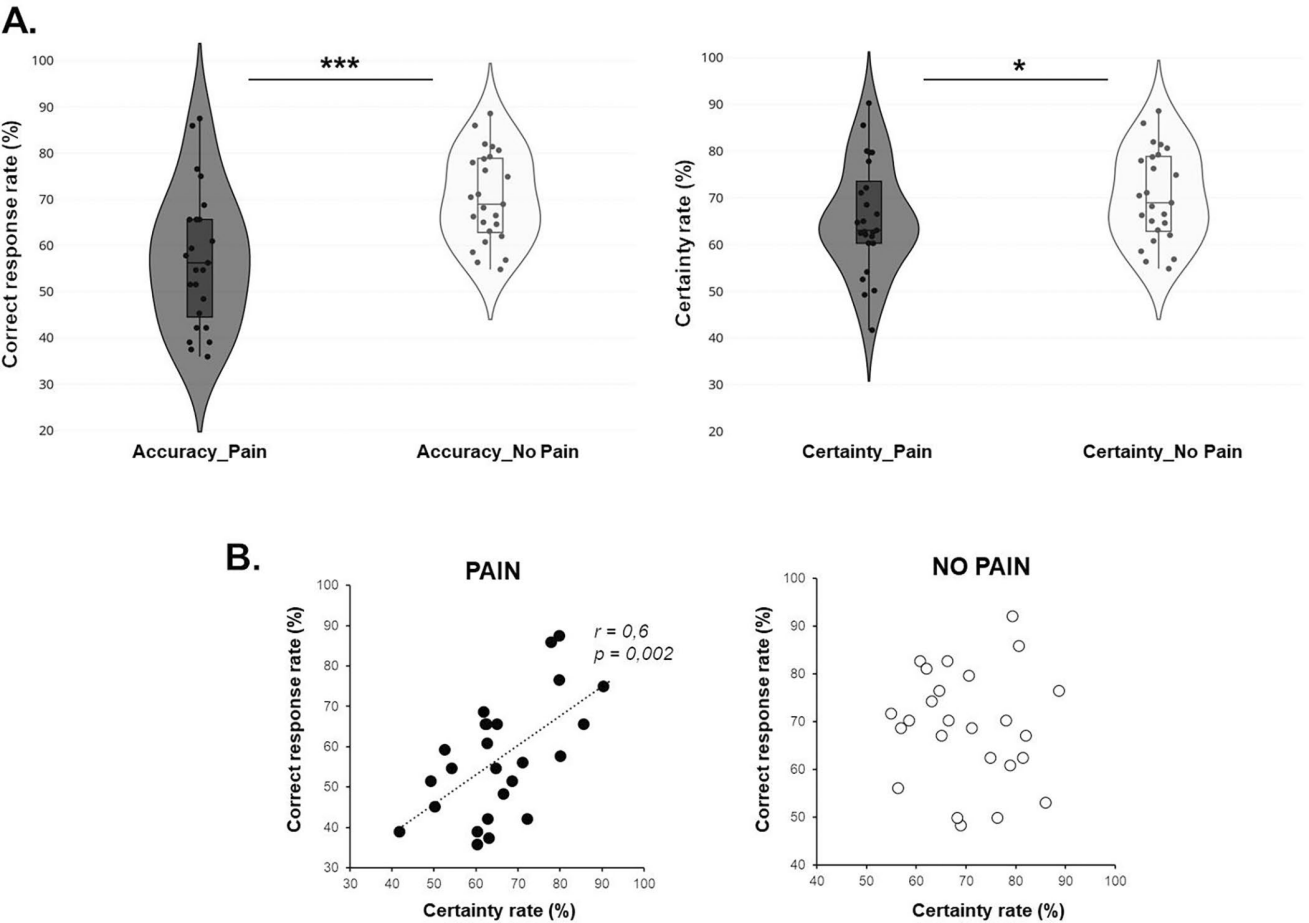
(A) Violin plots displaying the correct response and certainty rates in both painful and non‐painful modalities. ***p < 0.001, *p < 0.05 (B) Pearson correlations between accuracy (correct response rate) and certainty in the pain and no‐pain conditions. Each dot represents a single participant's performance. The dotted line indicates the best linear fit (least squares regression). Pearson's correlation coefficient (r) and corresponding *p*‐value are reported for significant correlation.

The correct response rates were of 57.31% ± 14.6% for painful stimuli and 69.22% ± 11.8% for the non‐painful ones. Paired‐sample *t*‐tests revealed that participants were significantly more accurate in the no‐pain condition compared to the pain condition (*t*(24) = −4.3, *p* < 0.001, Cohen's *d* = −0.9). High certainty rates were observed in each condition (65.78% ± 11.8% for painful stimuli, 70.4% ± 9.7% for non‐painful stimuli), also with an effect of stimulation type, subjects being more confident in their answers for non‐painful relative to painful stimuli (*t*(24) = −2.9, *p* < 0.05, Cohen's *d* = −0.6; Figure 3A).

A significant positive correlation was found between correct response rates and certainty in response to painful stimuli (*r* = 0.6, *p* = 0.002), suggesting that participants' confidence was better aligned with their performance under nociceptive conditions (Figure 3). In contrast, no significant correlation was observed in the no‐pain condition (*r* = −0.07, *p* = 0.7), indicating a possible dissociation between subjective certainty and actual performance when pain was absent (Figure 3B).

#### 3.2.2 Effect of Perceptual Difference Magnitude

The LMM analysis revealed that differences in VAS (ΔVAS) between the two stimuli in a pair significantly predicted memory performance (*p* = 0.002), indicating that greater discriminability was associated with more reliable memory encoding. The relationship between discriminability and memory performance did not differ between painful and non‐painful conditions (interaction non‐significant, *p* = 0.481). When discriminability (ΔVAS) was included as a covariate, the main effect of Condition (Pain vs. No‐Pain) no longer reached statistical significance (*F*(1, 35.7) = 2.41, *p* = 0.130), suggesting that the difference in perceptual discriminability between modalities was determinant to the observed performance gap.

#### 3.2.3 Effect of Inter‐Stimulus Interval (ISI)

**FIGURE 4:**
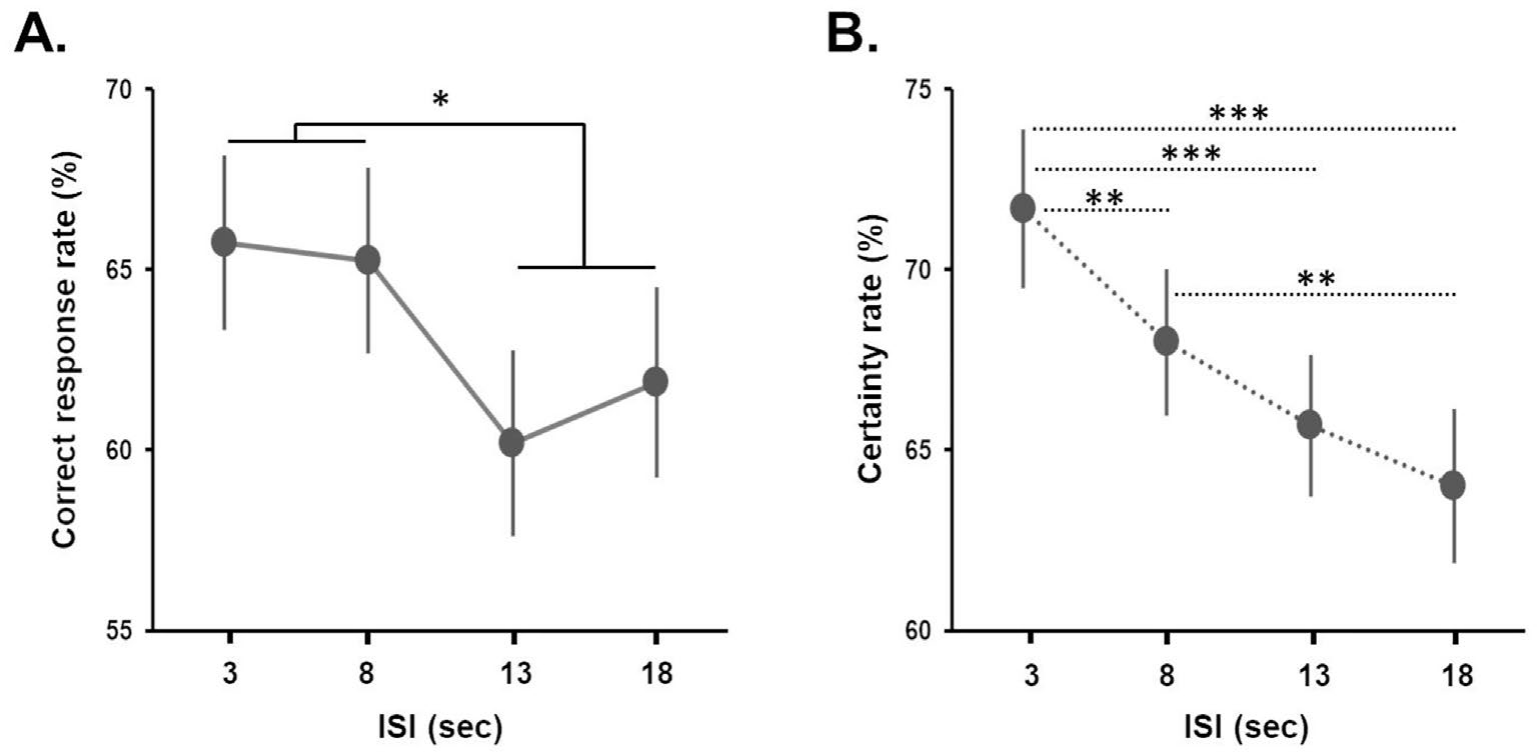
Effect of the ISI on the correct response (A) and certainty rates (B) for all stimuli (mean ± SEM). *p < 0.05; **p < 0.01; ***p < 0.001.

A significant effect of inter‐stimulus interval (ISI) was observed on correct response rates (*F*(2.7, 66.1) = 3.9, *p* = 0.015, partial *η* ^2^ = 0.14), with higher accuracy when the two stimuli were separated by shorter intervals (3 or 8 s), compared to longer intervals (13 or 18 s; Helmert contrasts, *p* < 0.05; Figure 4A). This effect was independent of stimulation type, as no significant ISI × condition interaction was found (*F*(2.4, 57.4) = 0.73, *p* = 0.51, partial *η* ^2^ = 0.03). To further examine whether the rate of temporal decay differed between modalities, we computed individual regression slopes of accuracy over ISI for each condition. Mean slopes were −0.41% ± 1.16%/s for nociceptive and −0.26% ± 1.02%/s for non‐nociceptive stimuli. A paired *t*‐test revealed no significant difference between conditions (*t*(24) = −0.41, *p* = 0.688, Cohen's *d* = −0.08), confirming that the rate of decay was comparable across modalities.

Certainty ratings also significantly declined as ISI increased (*F*(2.6, 63.2) = 21.7, *p* < 0.001, partial *η* ^2^ = 0.47), with no interaction between ISI and stimulation type (*F*(2.4, 56.3) = 0.19, *p* = 0.86, partial *η* ^2^ = 0.008), indicating a similar pattern across conditions. Bonferroni‐corrected post hoc comparisons showed that certainty was significantly higher at the 3‐s ISI compared to all other intervals (3 vs. 8 s: *p* = 0.0156; 3 vs. 13 and 18 s: *p* < 0.0001), and that certainty continued to decline between 8 and 18 s (*p* = 0.0018; Figure 4B).

#### 3.2.4 Effect of the Direction of Intensity Change

**FIGURE 5:**
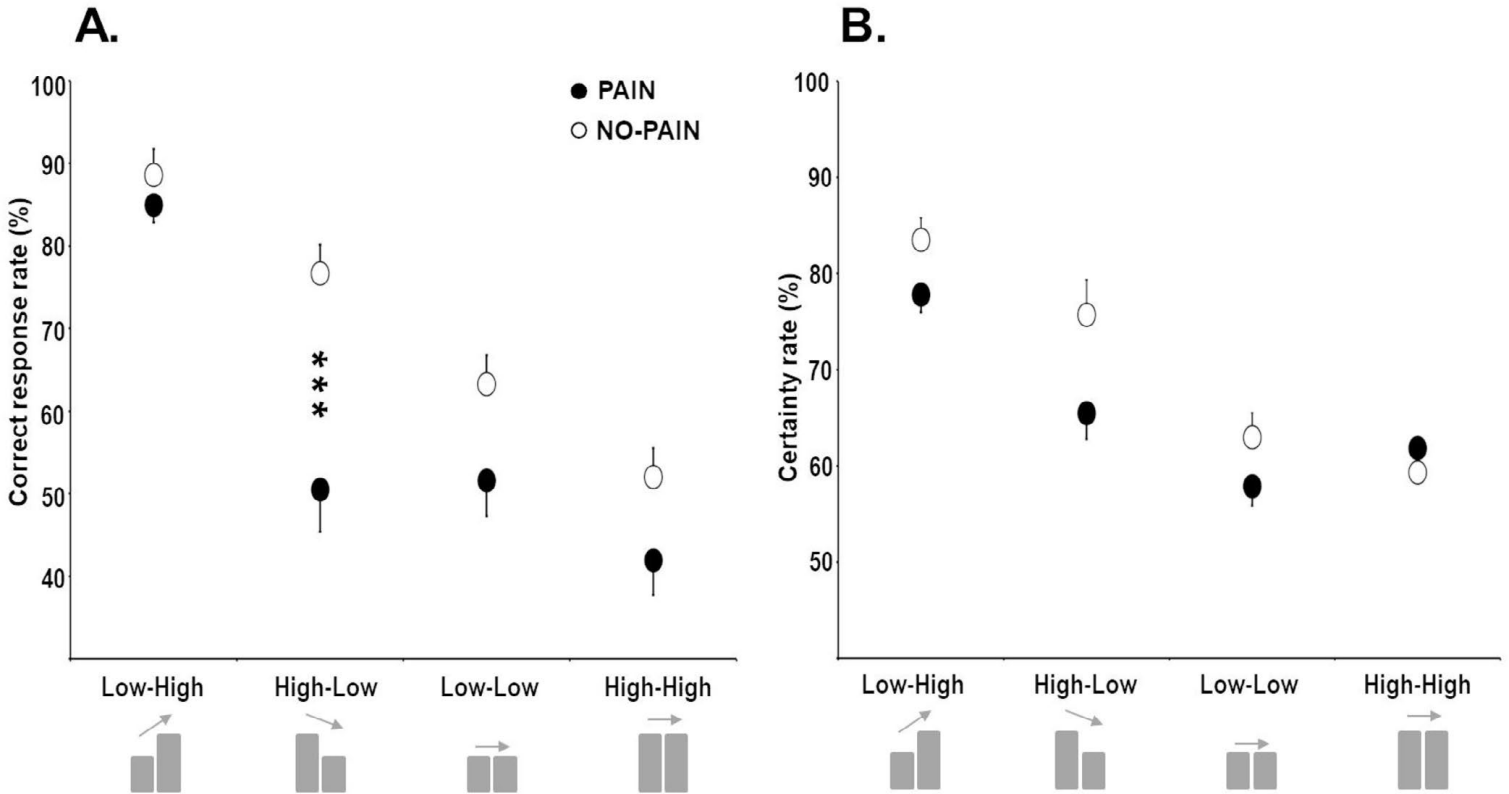
Effect of the pair of stimuli delivered on the correct response (A) and certainty rates (B) for painful (black dots) and non‐painful (white dots) stimuli (mean ± SEM). *p < 0.05; **p < 0.01; ***p < 0.001.

The relative intensity configuration of stimulus pairs (i.e., equal, ascending, or descending) significantly influenced the rate of correct detection (*F*(2.6, 62.3) = 40.9; *p* < 0.001; partial *η* ^2^ = 0.63). For both nociceptive and non‐nociceptive conditions, detection accuracy was highest when the second stimulus was more intense than the first (Low‐High pair; all Bonferroni‐corrected post hoc comparisons, *p* < 0.001). In contrast, performance was poorest when both stimuli were of high intensity, with significantly lower accuracy compared to other configurations (Bonferroni‐adjusted comparisons, *p* < 0.01 for all; Figure 5A).

**FIGURE 6:**
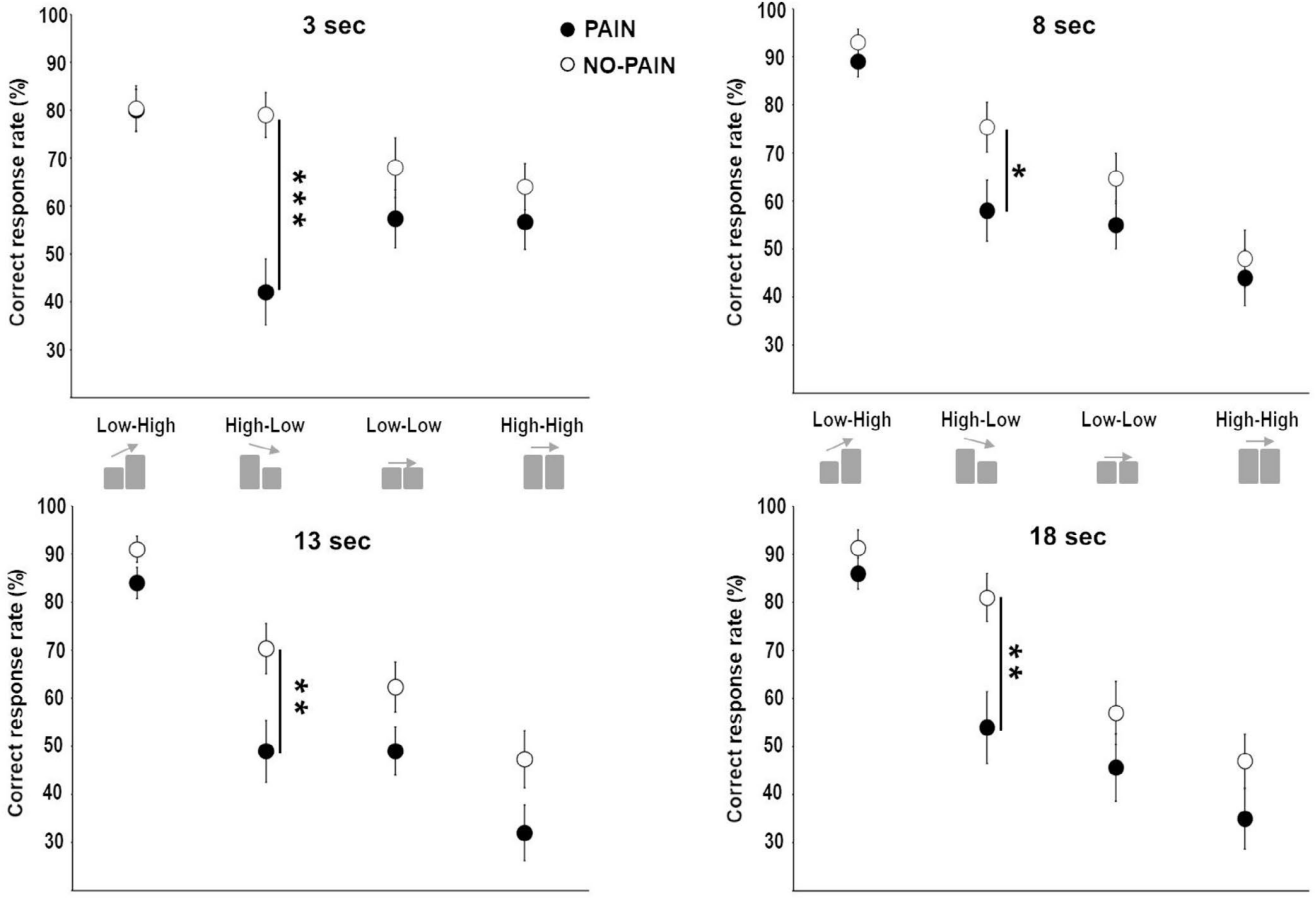
Effect of the pair of stimuli delivered on the correct response rates for each ISI for painful (black dots) and non‐painful (white dots) stimuli (mean ± SEM). *p < 0.05; **p < 0.01; ***p < 0.001.

ANOVA on correct response rates also revealed a significant interaction between stimulus pair configuration (high‐low, equal, low‐high) and stimulation type (painful vs. non‐painful; *F*(2.07, 49.6) = 8.15, *p* < 0.001, partial *η* ^2^ = 0.25). As illustrated in Figure 5A, this interaction was primarily driven by a marked decrease in accuracy for nociceptive stimulus pairs presented in a high‐to‐low intensity sequence (*t*(24) = −5.4, *p* < 0.001, Cohen's *d* = −1.07), a pattern that persisted across all inter‐stimulus intervals (Figure 6).

Certainty rates also varied as a function of the relative intensity of stimuli within a pair (*F*(2.38, 52.5) = 23.61, *p* < 0.001, partial *η* ^2^ = 0.52). Participants reported significantly greater confidence when the second stimulus differed in intensity from the first (Helmert contrasts, *p* < 0.001), with the highest certainty observed when the second stimulus was more intense than the first (Bonferroni‐corrected comparisons, *p* < 0.01 for all; Figure 5B).

ANOVA further revealed a significant interaction between stimulus pair configuration (high–low, equal, low–high) and stimulation type (painful vs. non‐painful; *F*(2.4, 52.8) = 5.79, *p* = 0.003, partial *η* ^2^ = 0.21). This interaction indicated that, for pairs with differing intensities, participants were more confident in their responses during non‐nociceptive trials. Specifically, confidence was higher for non‐painful compared to painful stimuli in both low–high (*t*(24) = −2.4, *p* < 0.05, Cohen's *d* = −0.5) and high–low conditions (*t*(24) = −3.2, *p* < 0.01, Cohen's *d* = −0.6). In contrast, for pairs of equal intensity, certainty ratings remained low regardless of stimulus type (low–low: *t*(24) = −1.9, *p* = 0.07, Cohen's *d* = −0.38; high–high: *t*(24) = 1.2, *p* = 0.22, Cohen's *d* = 0.2; Figure 5B).

#### 3.2.5 Analysis of Errors: Over‐ Versus Under‐Estimates

**FIGURE 7:**
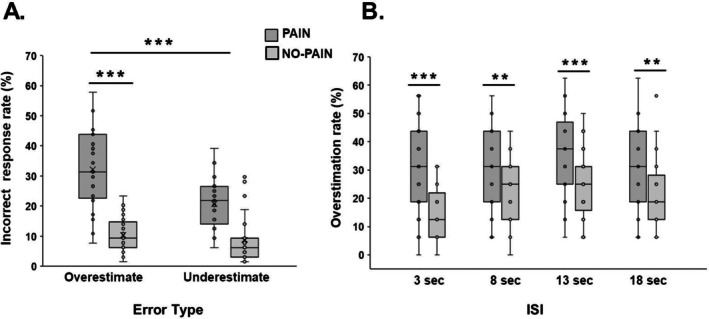
(A) Global overestimate and underestimate means for painful and non‐painful stimuli. (B) Overestimation rates for painful and non‐painful stimuli depending on ISI. **p ≤ 0.01, ***p < 0.001.

Incorrect responses could reflect either an overestimation or an underestimation of the second stimulus relative to the first. The ANOVA revealed a significant main effect of error type (*F*(1, 24) = 58.17, *p* < 0.001, partial *η* ^2^ = 0.71) indicating that participants generally produced more overestimations than underestimations across all stimulation types (Bonferroni‐corrected comparisons, *p* < 0.001). A significant interaction was also found between stimulus type (nociceptive vs. non‐nociceptive) and error type (over‐ vs. underestimation; *F*(1, 24) = 23.84, *p* < 0.001, partial *η* ^2^ = 0.49). Specifically, participants overestimated the second stimulus more frequently in response to painful than to non‐painful stimuli (*t*(24) = 5.9, *p* < 0.001, Cohen's *d* = 1.18) whereas underestimation rates did not differ between stimulus types (*t*(24) = 0.9, *p* = 0.36, Cohen's *d* = 0.19; Figure 7A).

The highest rate of overestimations for painful stimuli was consistent across all ISIs (3 s: *t*(24) = 5.2, *p* < 0.001, Cohen's *d* = 1.04; 8 s: *t*(24) = 3.4, *p* = 0.002, Cohen's *d* = 0.68; 13 s: *t*(24) = 3.99, *p* < 0.001, Cohen's *d* = 0.8; 18 s: *t*(24) = 2.79, *p* = 0.01, Cohen's *d* = 0.56; Figure 7B).

**FIGURE 8:**
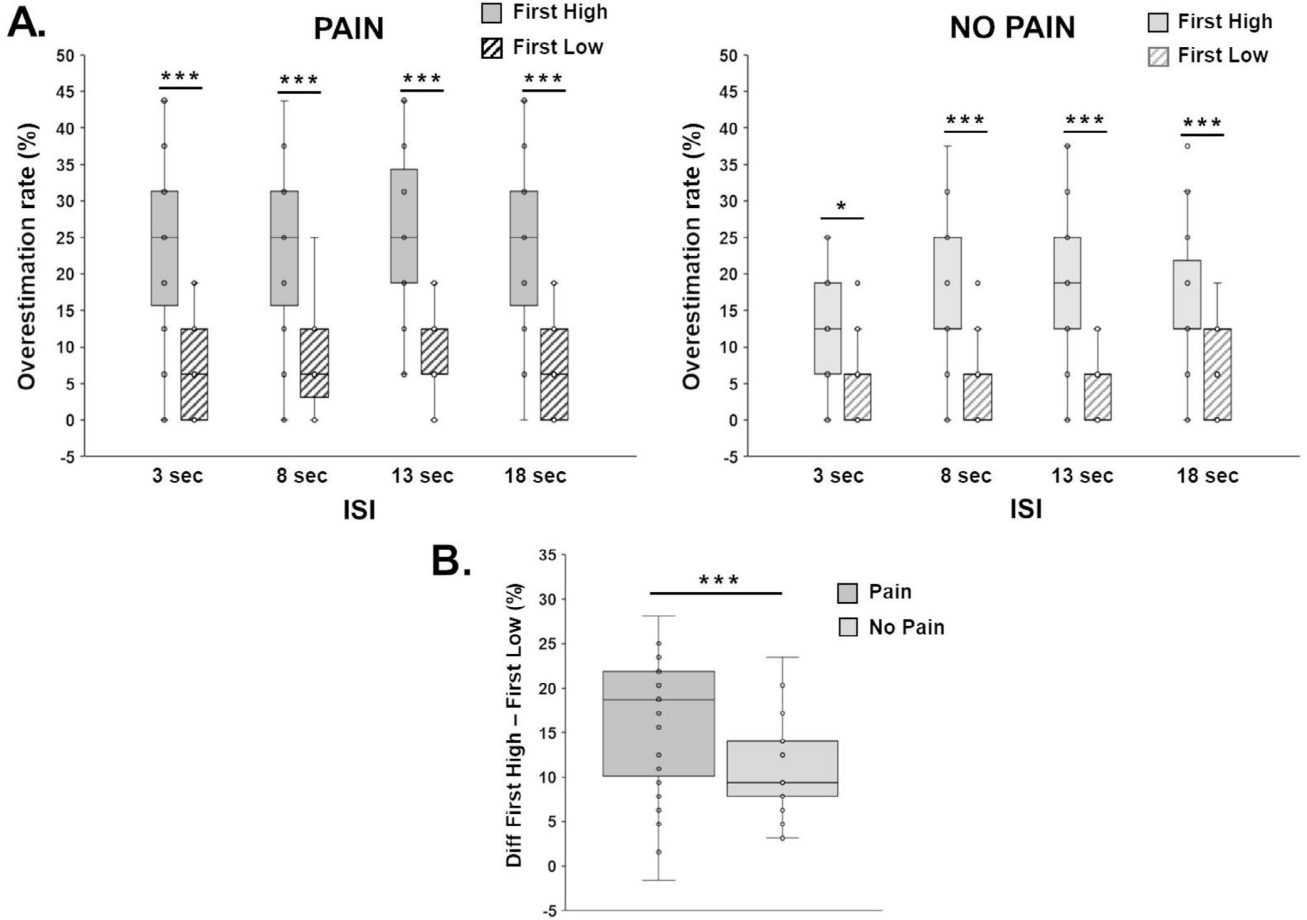
(A) Overestimation rates for painful and non‐painful stimuli depending on pair of stimuli delivered (First High vs. First Low) and ISI. (B) Differences in the overestimation rates after high or low intensity stimulation for painful or non‐painful stimuli, regardless of ISI *p < 0.05, ***p < 0.001.

Overestimation rates were also modulated by the relative intensity of the stimulus pairs. When the first stimulus was of higher intensity, participants were more likely to overestimate the second one, both for painful (*F*(1, 24) = 103.04, *p* < 0.001, partial *η* ^2^ = 0.81) and non‐painful stimuli (*F*(1, 24) = 74.6, *p* < 0.001, partial η^2^ = 0.76), regardless of the ISI (Figure 8A). While this pattern was observed in both conditions, the effect was significantly stronger for painful stimuli, the difference in overestimation rates for pairs of stimuli beginning with high versus low intensity being greater for nociceptive shocks (*t*(24) = 3.7, *p* < 0.001, Cohen's *d* = 0.71; Figure 8B).

To determine whether the observed overestimation bias was specific to conditions requiring active memorization of stimulus intensity, a control condition was implemented. In this condition, stimuli of either low or high intensity were presented in a randomized order, and participants were instructed to rate each stimulus immediately using a visual analog scale (VAS), without any memory demand (see Methods). Participants accurately rated stimulus intensities, and these ratings were not influenced by the preceding stimulus. Specifically, no significant differences were found in VAS scores for low‐intensity stimuli delivered after either a low or a high stimulus (painful: 49.50% vs. 50.06% of VAS maximum; non‐painful: 30.78% vs. 29.08%; all *p* > 0.05), nor for high‐intensity stimuli preceded by either a high or a low stimulus (painful: 70.63% vs. 78.53%; non‐painful: 77.75% vs. 82.32%; all *p* > 0.05). These results indicate that the overestimation bias did not occur when no retention or comparison process was required.

#### 3.2.6 Visual “Match‐To‐Sample” Memory Task

This task was interleaved between each block of “memory for intensity” trials to minimize potential residual memory effects and to prevent prior intensity comparisons from influencing subsequent judgments of somatosensory stimulus pairs (see Methods). Performance on the visual task was consistently high (mean accuracy = 93% ± 4.14%) and did not vary significantly across the four experimental sessions (*F*(3, 24) = 1.397, *p* = 0.25, partial *η* ^2^ = 0.05), indicating that participants maintained stable attention throughout the experiment.

## 4 Discussion

The aim of the present study was to compare short‐term memory for nociceptive and non‐nociceptive somatosensory stimuli using a delayed discrimination paradigm, focusing on retention accuracy, temporal decay, perceptual biases and subjective confidence. While a number of features were common to the encoding of noxious and non‐noxious stimuli, others appeared to differentiate both modes of encoding. Both aspects are discussed in what follows.

### 4.1 Reduced Accuracy and the Role of Perceptual Discriminability

There was a robust difference in overall memory accuracy, which was significantly lower for nociceptive than non‐nociceptive stimuli (57% vs. 69%, *p* < 0.001), despite a similar subjective confidence reported for both modalities. A key contributing factor to this performance gap was the difference in perceptual discriminability between intensity levels: the mean ΔVAS (difference in VAS for low‐ and high‐intensity stimuli) was smaller in the nociceptive condition (~29.7 units) than in the non‐nociceptive condition (~42.4 units), and when ΔVAS was included in linear models as a covariate, it accounted for the main effect of modality on accuracy. This result is fully consistent with the Weber‐Fletcher's law, establishing that the just‐noticeable difference between two stimuli increases proportionally to the actual magnitude of the stimuli. In practical terms, this means that two stimuli separated by the same intensity step are harder to discriminate when they are both of high intensity. This inherent limitation of intensity coding at high magnitudes may contribute to inaccurate pain recall and comparisons in clinical settings, since comparing past relative to present pain operates in the high‐intensity range of the somatosensory scale, where perceptual resolution and memory accuracy are lowest.

Of notice, even the performance level for *non‐nociceptive* stimuli, ~70% in our subjects, was slightly lower than that previously reported for other types of non‐noxious somatosensory input, which could reach 85%–90% at comparable intervals for vibratory (Bigelow and Poremba 2014) or warm stimuli (Rainville et al. 2004). A critical difference between these previous reports and the present study is the duration of target stimuli, which reached several seconds for vibratory or warm stimuli previously reported, but less than 1 millisecond in the present study. Longer stimulus durations authorise a more robust encoding in short‐term memory buffers (Ratcliff et al. 1990; Caplan and Guitard 2024) and likely explain the slight disadvantage in the present experiments. Although highly speculative, one might wonder whether the impact of stimulus duration on the encoding of pain memories could be extrapolated to clinical situations, since for instance the duration of initial acute pain is thought to influence its chronification (Althaus et al. 2014; Borsook et al. 2018).

A number of differential results between noxious and innocuous stimulus coding could not be attributed to differences in discriminability alone. These include a specific directional bias in accuracy, a selective overestimation pattern, and differential dissociation between confidence and accuracy, all suggesting that the nociceptive quality itself may impose constraints on memory encoding and maintenance, beyond what can be explained by sensory discriminability.

### 4.2 Temporal Decay

Memory performance declined systematically as the inter‐stimulus interval increased, and this was similarly observed for both nociceptive and non‐nociceptive pairs of stimuli. This is consistent with findings from visual, auditory, and tactile short‐term memory, where performance decreases over time even in the absence of interfering stimuli (Bigelow and Poremba 2014; Serences 2016). Such temporal decay, typically attributed to attentional fluctuations and neural noise accumulating over the retention interval (Adam and deBettencourt 2019; Ricker et al. 2016; Ueda et al. 2022), was equivalent for nociceptive and non‐nociceptive stimuli, as shown by direct comparison of individual regression slopes of accuracy over ISI. This aligns with earlier work by Rainville et al. (2004), who showed a very similar temporal decay of memory representations for warm and noxious heat, using similar time intervals as in the present study. Our results expand and generalise this earlier work to stimuli of very different nature, both in modality (electrical vs. thermal) and duration (< 1 msec vs. 2–6 s). Taken together, while nociceptive memory traces consistently appear less accurate overall, they do not degrade more rapidly than non‐nociceptive traces, at least over the 3–18 s window examined here and in Rainville's study.

### 4.3 Directional Encoding Bias: An Asymmetry Specific to Nociception?

For both noxious and non‐noxious pulses, the proportion of correct responses and associated subjective certainty were highest when the second stimulus was more intense than the first. This pattern might reflect the greater signal‐to‐noise advantage conferred by an ascending intensity step relative to an equal or descending one, since it is known that noise tends to increase short‐memory bias (e.g., Olkkonen et al. 2014; Ueda et al. 2022). A second stimulus that exceeds the retained trace of the first may additionally benefit from a contrast‐facilitation effect, whereby a physically salient change relative to the memory trace enhances detection accuracy.

A concurrent *dis‐facilitation* pattern emerging when the intensity of the 2nd stimulus fell below the first (high‐low stimulus pairs) was markedly different for non‐painful and painful stimuli, the latter showing a much higher decline in accuracy, which dropped to levels comparable to low‐low intensity trials across all intervals (Figure 6). This directional encoding bias suggests that the short‐term representation of nociceptive intensity was particularly sensitive to the direction of change, with high–low transitions being disproportionately difficult to encode or retrieve correctly. One possible interpretation is that an intense initial nociceptive stimulus sets a high reference level that biases the encoding of a subsequent, weaker stimulus upward, a form of anchoring effect that was either absent or substantially weaker for our non‐nociceptive stimuli. This directional asymmetry cannot be attributed to discriminability differences, since the absolute perceptual contrast between high and low intensities was the same regardless of presentation order.

### 4.4 Systematic Overestimation Bias: A Central Rather Than Peripheral Origin?

Participants were more likely to judge the second stimulus as more intense than it actually was, such overestimation being significantly more prevalent for painful than non‐painful stimuli across all intervals (Figure 7A,B). Overestimation errors mainly occurred when the first stimulus of a pair was of high intensity, and again this was significantly more prevalent for nociceptive stimuli, and explained the loss of accuracy in high‐low pairs discussed above. These data suggest that an intense initial nociceptive stimulus not only degrades the accuracy of a subsequent comparison, but actively distorts it in a directional manner. Critically, this overestimation was entirely absent when participants were asked to rate each stimulus in a sequence immediately upon delivery, without any retention or comparison requirement. Under these conditions, ratings were unaffected by the intensity of the preceding stimulus, suggesting that the bias did not arise from stimulus‐related factors (e.g., peripheral sensitization) but was related to processes recruited during the encoding, maintenance, or retrieval of nociceptive intensity. Sensitization could also be ruled out since the overestimation bias was consistent and virtually identical across all ISIs from 3 to 18 s, whereas peripheral sensitization effects would be expected to prevail at short stimulus intervals.

### 4.5 Confidence–Accuracy Dissociation

While certainty ratings were well above 65% in both conditions, accuracy was on average 12% points lower for nociceptive input. This means that participants performing at chance or near‐chance level on nociceptive trials, often overestimating the intensity of the stimulus, nonetheless reported moderate to high confidence in their responses. Transposed to clinical settings, this dissociation reminds the common observation that “confident memories” of past pain reported by patients are most often inaccurate when compared to actual daily pain rates (Matera et al. 2003; Daoust et al. 2017; Wikström et al. 2017), with recall reports being often inflated relative to momentary or daily ratings (Broderick et al. 2008; Giske et al. 2010; Daoust et al. 2017).

## 5 Limitations

Several limitations should be acknowledged. Our modest sample size (*n* = 25), while sufficient to detect robust effects, may have hindered the detection of subtle individual differences. In addition, the use of brief electrical stimuli (100 μs) prioritized experimental control over clinical or ecological validity. While allowing precise temporal manipulation and minimizing peripheral adaptation, these stimuli bear limited resemblance to clinical pain—typically longer, more complex, and emotionally embedded. Consequently, the present findings primarily inform fundamental mechanisms of nociceptive memory rather than directly modelling clinical pain memory.

## 6 Conclusion

This study shows that short‐term memory for acute nociceptive stimuli is less accurate, more directionally biased, and more prone to systematic overestimation than memory for non‐nociceptive somatosensory input—even over timescales of a few seconds. The performance gap may be largely explained by reduced perceptual discriminability in the nociceptive range, a direct consequence of the Weber–Fechner compressive relationship with practical implications for clinical pain intensity assessment. Beyond perceptual factors, three modality‐specific distortions: a directional encoding asymmetry favouring ascending over descending pairs; a systematic overestimation bias of central origin, and a confidence–accuracy dissociation reveal additional constraints imposed by nociceptive processing on memory encoding and maintenance. These findings provide a psychophysical basis for understanding pain memory distortions in clinical populations and highlight the inherent vulnerability of short‐term nociceptive memory traces.

## Author Contributions

This study was designed by M.F. and L.G.‐L. The experiments were performed by M.F., C.P. and J.G. The data were analysed by M.F. and C.P. Additional analyses in response to reviewer feedback were conducted by M.F. The results were critically examined by all authors. M.F. had a primary role in preparing the manuscript, which was edited by C.P., J.G. and L.G.‐L. All authors have approved the final version of the manuscript and agree to be accountable for all aspects of the work.

## Funding

This work was supported by the APICIL Foundation.

## Conflicts of Interest

The authors declare no conflicts of interest.
